# An IoT-Based Smart System with an MQTT Broker for Individual Patient Vital Sign Monitoring in Potential Emergency or Prehospital Applications

**DOI:** 10.1155/2022/7245650

**Published:** 2022-01-29

**Authors:** Yung-Chung Tsao, Fu-Jen Cheng, Yi-Hua Li, Lun-De Liao

**Affiliations:** ^1^Institute of Biomedical Engineering and Nanomedicine, National Health Research Institutes, Zhunan, Taiwan; ^2^Department of Emergency Medicine, Kaohsiung Chang Gung Memorial Hospital, Kaohsiung, Taiwan; ^3^Department of Finance, Chung Yuan Christian University, Taoyuan City, Taiwan

## Abstract

Emergency care is a critical area of medicine whose outcomes are influenced by the time, availability, and accuracy of contextual information. The success of critical or emergency care is determined by the quality and accuracy of the information received during the emergency call and the data collected during emergency transportation. The Internet of Things (IoT) consists of many smart devices and components that communicate via their connection to the Internet, which is used to collect data with sensors that obtain personal health parameters. In the past, most health measurement systems were based on a single dedicated orientation, and few systems had multiple devices on the same platform. In addition to traditional health measurement technologies, most such systems use centralized data transmission, which means that health measurement data have become the exclusive intellectual asset of the system developer. Therefore, this study develops an IoT-based message-broker system that is deployed and demonstrated for five health devices: blood oxygen, blood pressure, forehead temperature, body temperature, and body weight sensors. A central controller accessed by radio-frequency identification (RFID) collects clients' health profiles on the cloud platform. All collected data can be quickly shared, analyzed, and visualized, and the health devices can be changed, added to, and removed reliably when the requirements change. Additionally, following the message queuing telemetry transport (MQTT) protocol, all devices can communicate with each other and be integrated into a higher-level health measurement standard (such as blood pressure plus weight or body temperature plus blood oxygen). We implement a smart healthcare monitoring system (SHMS) and verify its reliability. We use MQTT to establish an open communication format that other organizations can follow to perform individual patient vital sign monitoring in potential applications. The robustness and flexibility of this research can be verified through the addition of other systems. Through this structure, more large-scale health detection devices can be integrated into the method proposed in this research in the future. Personal RFID or health insurance cards can be used for personal services or in medical institutions, and the data can easily be shared through the mechanism of this research. Such information sharing will enable the utilization of medical resources to be maximized.

## 1. Introduction

The Internet of Things (IoT) is a novel network that connects ordinary devices or objects to the Internet, offering automatic machine-to-machine (M2M) communication without human interaction. This emerging technology provides upgrades and improves arrangements in the clinical application for suitable medical record-keeping, sampling, integrating devices, and determining the causes of possible diseases. The sensor-based technology of the IoT provides the excellent capability to reduce the risk involved in clinical treatment in complicated cases and has been helpful in the COVID-19 pandemic [1]. In the clinical field, researchers have focused on how to use the IoT to help precisely treat different COVID-19 cases [2, 3]. The IoT lightens the workload of medical doctors by minimizing risks and increasing overall performance. Doctors can use this technology to easily detect changes in the critical parameters of COVID-19 patients or patients in an emergency unit [4]. The information-based service opens up new healthcare opportunities by moving toward the best way for an information system to adopt new results and improve hospital treatment systems [5]. The proper usage of the IoT can help overcome different medical challenges, such as speed, price, and complexity [6, 7]. The IoT can easily be customized to monitor individual patients' vital signs and the treatment of COVID-19 patients [8, 9]. The overall performance of healthcare can be improved via a digitally controlled health management system during an emergency such as the COVID-19 pandemic [9].

According to recent research, the current number of IoT devices is greater than 50 billion, with explosive growth through 2020 and possibly beyond [10–12]. In IoT architecture approaches, an increasing number of ubiquitous IoT sensors are connected to devices that can be distributed anywhere and are connected to a cloud platform [13]. In the future, these approaches will shift the existing information system (IS) to a new stage as an increasing number of IoT applications are developed and deployed to promote intelligent healthcare, smart cities, smart transportation, smart grids, industrial automation, smart homes, etc. [14–18]. Among the abovementioned market domains of the IoT, innovative healthcare is likely the leading sector [13, 18–20]. With the global population increasing daily, the increasing number of senior citizens has introduced new critical challenges, resulting in a variety of societal problems [21, 22]. For example, increasing numbers of patients with chronic hypertension, diabetes, gout, heart disease, cancer, and cerebrovascular disease, as well as their family members, are suffering from traumatic conditions daily. If these individuals could select their health plans and their health indexes could be monitored by their attending physicians before and after they become sick, perhaps, these issues could gradually be reduced.

According to the World Health Organization (WHO), 2 billion people will be sixty years of age or older by 2020, and half of the developing world population will be prone to becoming chronically ill [23]. The cost of hospitalization and long-term care will continue to increase and be difficult to cover; thus, home care or personal health monitoring may provide a reasonable solution to reducing these expenses [24]. Daily health monitoring and reporting to family members and the National Health Insurance Administration can help us understand health indexes and client symptoms based on the use of long-term and continuous health parameters to obtain detailed diagnoses. Moreover, the IoT architecture of healthcare monitoring systems (HMSs) can address the connectedness of medical information systems (MISs) while ensuring the safety and confidentiality of patient information. In many cases, MISs have refused to join nonmedical ISs due to the heterogeneous hardware requirements and software complexity; instead, the choice is made to maintain the stability of the MISs [25, 26]. Therefore, many health information systems (HISs) have failed to be integrated into MISs.

Due to the issues noted above, making HMSs smarter will require easy sharing mechanisms and connection elasticity for patient-related health data. In contrast to patient privacy in an IS for general enterprises, patient privacy in an MIS has always been considered a critical factor under related medical laws and rules of medical treatment. Under these limitations, as previously mentioned, it is challenging and sometimes impossible to develop common ways to connect to several MISs with a unified standard system specification. However, the IoT architecture may overcome this bottleneck based on the foundation of data-oriented approaches, and a public data hub could be established as middleware with which anyone could send and receive data. Any IS could decide when, how, and what to send and receive instead of instantly adjusting its approach to fit all other ISs [27]. This simple method reverses the roles of data requesters and data dispatchers, simplifying the ability to connect to others [28].

A remote monitoring mechanism for patients and their family members is urgently needed in HISs, even though the delivery of automatic notifications and warnings to family members is currently the most basic requirement of an HIS [29]. Smart healthcare should be easily accessible and shared in community health facilities and care centers. With the current issue of the global aging population, high traffic costs, and the high cost of medicine, rapid online diagnosis has become an urgent need for many clients. An IoT-based IS implemented in the healthcare field could have great practical value in providing online reports that can be visualized and analyzed as charts by patients and their family members when these users access the cloud platform. Online services could summarize these personal health reports according to the relevant health parameters, based on a rule-based repository, and send a long-term health report to the attending physician that can easily be integrated into the corresponding MIS; the corresponding information would then be actively received by other supported ISs [30, 31].

To achieve these goals, effective middleware for data exchange must be built for MISs. Such middleware would provide an indispensable kernel function to support scalable and reliable connections to other ISs, thus building a smart bridge to external MIS and IoT applications with high efficiency and reliability in operations and data exchange based on each IS. These applications could choose to access or reject transmissions from the data hub instead of establishing mutual bonds on the basis of individual development schedules. Open and standard middleware types for outer connections based on IoT technology, such as representative state transfer (REST) application programming interfaces (APIs), constrained application protocols (CoAPs), message queuing telemetry transport (MQTT) services, data distribution services (DDSs) for real-time systems, advanced message queuing protocols (AMQPs), extensible messaging and presence protocols (XMPPs), the Java message service (JMS), and simple object access protocols (SOAPs), are popular and easily deployable.

Based on the issues noted above, an open and smart healthcare system that includes a standard data exchange format and biocommunication system specifications is developed in this study to solve the problems associated with establishing a bridge to MISs and to encapsulate sensors in distributive and deployable IoT-based devices with external communication abilities [32]. This novel approach will overcome the current closeness problems in MISs and satisfy the abovementioned criteria and other critical requirements.

In this study, we aim to design and implement a smart healthcare IS with an IoT-based architecture (referred to as a smart healthcare monitoring system (SHMS)) to measure and send heart rate, blood oxygen level, blood pressure, body weight, and continuously monitored body temperature and thermal temperature information to the cloud platform using REST APIs via the Internet. In addition, we design a human-machine interface (HMI) device referred to as a radio-frequency identification (RFID) controller, which can be accessed by RFID cards such as health ID cards and student cards with embedded RFID information to identify users' names (to retrieve the associated profiles) and to access other health devices via the MQTT protocol. Additionally, user profiles can be published and edited with the collected information to instantaneously retrieve various results. This advanced technique can dynamically select different types of health devices (for the same measurement variable) if other health devices are registered on the cloud platform. The developed SHMS can overcome the limitations of fixed health measurement variables and existing health devices, which can be replaced with home-use sensors, commercial sensors, or even Food and Drug Administration (FDA)-approved sensors by adding control and communication layers to the sensors without redesigning them or performing mass migration. With this proposed mechanism, the SHMS enables the addition, removal, exchange, and migration of health devices to enhance system scalability and usability, enabling non-MISs to join an MIS without heterogeneous hardware and software complexity issues while maintaining system stability. We plan to deploy the SHMS through the Internet for user testing and validation over a six-month period to verify the effects after we achieve the abovementioned goals. Of course, the SHMS will be modified and upgraded based on user comments to ensure that all efforts and functions facilitate the realization of the relevant goals.

## 2. Materials and Methods

### 2.1. System Architecture

**Figure 1:**
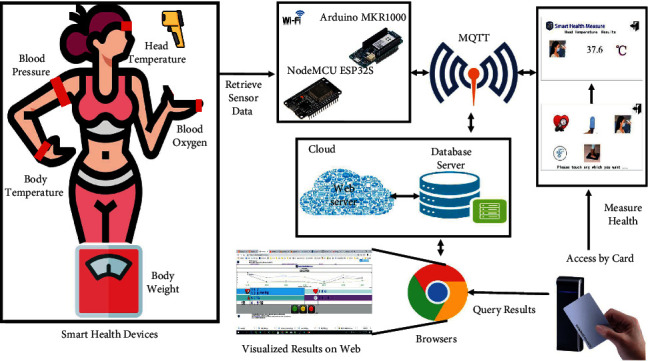
Proposed system architecture, including the layout and system structure, how each device (developed with an MCU, including an ESP32 and an Arduino MKR1000) measures clients' physiological health parameters and transmits sensor data via an MQTT broker to the cloud platform (which is established in this research), and how all data are stored in a database server that is accessible via a website. Users can instantly obtain any measured result on an RFID device after taking a measurement, including head temperature, body temperature, body weight, blood oxygen level, and blood pressure. In particular, users can obtain integrated online health reports through a browser via a PC, smartphone, etc. Furthermore, all information can be collected and classified into personal health records via health ID cards and synchronously shared with family members and physicians. This proposed system architecture facilitates information sharing (isolated from developers' technology barriers and gaps) based on pure information flows through an MQTT broker despite using individual information technology (IT) structures designed and developed by each enterprise or organization.

This study proposes a novel and distributed architecture based on the information-flow approach to simplify data sensing, data analysis, data visualization, and user profiles, as shown in Figure 1. The figure presents a smart healthcare system developed by using ESP32 connected to an MQTT broker with the MQTT protocol via the Internet. The MQTT protocol is an advanced and convenient technology that was created by IBM in the 1990s. It is based on an asynchronous messaging protocol, which decouples the message sender and receiver, in both space and time. It is scalable in unreliable network environments and sends and receives simple commands with moderate-sized content under a certain quality of service (QoS) that is guaranteed globally [33]. Hence, the transition from legacy systems to cloud platforms is possible without redesign or mass modification, which requires considerable effort and cost. These integrations can also be performed in the future.

Smart healthcare systems combine many smart health devices (which are designed with many sensors, such as pulse rate sensors, SpO_2_ sensors, contactless infrared thermometers, highly precise thermocouplers used as temperature sensors, and load cell sensors) for physiological data retrieval. The system architecture allows new health devices to join the SHMS despite heterogeneous hardware or software types. We apply object-oriented approaches to encapsulate the hardware and software components as an object with an abstract outer interface for different connections. IS infrastructure innovations have rapidly expanded and have led to the use of many system structures, such as mass-distributed systems, cloud computing, edge computing, fog computing, and the industrial IoT, in IoT applications. Therefore, the central criteria of the SHMS proposed in this paper should be related to the corresponding information-flow approaches; this approach can enhance our system and allow devices to communicate via an MQTT broker without changing the hardware, software, or design methodology. The advantages of the SHMS also facilitate the development of the proposed system as a component-block-stacked and message-oriented middleware (MOM) architecture. This architecture eliminates the gaps between hardware/software and software processes with unique methodologies, software development processes, and hardware/software compatibilities. Additionally, all devices can collaborate with a messaging flow by publishing with and subscribing to the MQTT broker, as shown in Figure 1. The IoT-based SHMS integrates a cloud platform visualized in the style of a web service as a central system and dispatches measurement jobs to distributed health devices with RFID tags delivered through distributed systems (subsystems or health devices).

### 2.2. Procedural Steps

**Figure 2:**
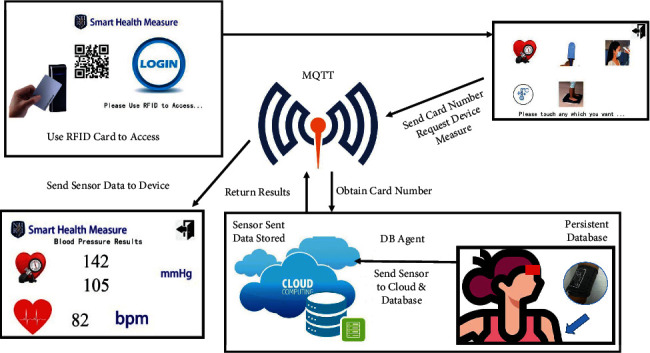
Workflow of the system proposed in this paper. Step 1: users use their health ID card to access the RFID controller, which can read the RFID inner ID number, and choose measurement options on a TFT screen to select the measurement device. Step 2: the RFID controller sends a health ID card and job ID via an MQTT broker over a Wi-Fi connection to notify the selected device (head temperature, body temperature, body weight, blood oxygen, or blood pressure sensor) to be ready for use. Step 3: users use the selected device, which then sends results via the MQTT broker and cloud platform over a Wi-Fi connection. All results are instantly displayed on both the TFT touch screen of the RFID controller and a browser running on any smartphone or desktop PC.

The goal of the proposed system is to collect health data from the health devices and send these data to the cloud platform, as shown in Figure 2. Each user must use an RFID card similar to a health ID card or student card embedded with RFID information to access the RFID controller, as shown in the upper-left box in Figure 2. In the first step, the RFID controller obtains the RFID tag ID after the RFID reader is accessed with an RFID card; then, the RFID controller obtains an ID number and displays it on the thin-film transistor (TFT) screen. In the second step, the user touches the login icon on the main screen of the SHMS and enters the health device menu, as shown in the upper-right box in Figure 2, to select the variable to be measured. In the third step, the RFID controller sends the tag ID and job commands via the MQTT broker to be published for a specified topic to indicate which health device should be activated for the specified user. In the fourth step, the specified user uses the assigned health device to retrieve his or her health measurement, as shown in the lower-right box in Figure 2. The assigned health device sends all results to the database (DB) agent on the cloud platform for long-term storage. The health device also sends the same results via the MQTT broker to be published, and the RFID controller is notified. The health information is visualized on the TFT screen, as shown in the lower-left box in Figure 2, and the specified user is notified. A blood pressure measurement is shown in Figure 2 as an example.

**Figure 3:**
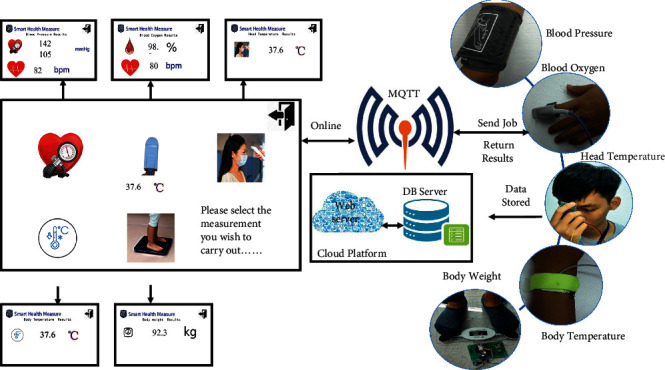
Operational flow implemented by users (checking essential vital signs) in the system proposed in this paper. Step 1: users access the RFID controller with their health ID card and select the measurement option on the TFT touch screen. The RFID controller sends the corresponding ID number via an MQTT broker over a Wi-Fi connection to notify the above devices (head temperature, body temperature, body weight, blood oxygen, and blood pressure sensors) to be ready for use. Users can use any of the above devices to detect their health status, after which the devices will send results via the MQTT broker and cloud platform over a Wi-Fi connection. All results are instantly displayed, either individually or in summary, on the TFT touch screen of the RFID controller and on any browser running on a smartphone or desktop PC.

The SHMS integrates five health devices to measure the corresponding health indexes. We can add new health devices to the proposed architecture to meet new requirements, but these five health devices—head temperature, body temperature, body weight, blood oxygen, and blood pressure sensors—are currently sufficient for our clients. Users can operate any of the five health devices to measure the corresponding health index according to the medical advice of his or her doctor or nurse regarding continuous monitoring, as shown in Figure 3. The operational procedures for the five health devices are almost identical, as shown in Figure 2. Continuous monitoring of the research goals can meet doctors' needs for patient assessment, and body temperature, pulse rate, SpO_2_ percentage, and body movement can be tracked. To achieve the abovementioned goals, we apply two kinds of powerful microcontrollers (MCUs), an ESP32 (ESP32 Series DevKits; Espressif Systems (Shanghai) Co., Ltd., China) and an Arduino MKR1000 (Arduino MKR1000; Genuino, USA), to promote hardware and software scalability and compatibility.

For an IoT-based system, Wi-Fi or wireless networking capability is required; additionally, the MCU needs to support a Wi-Fi connection, and the driving sensor needs to sense and retrieve feedback data. Consequently, a very large memory and high processing speed are required. To connect to the Internet by Wi-Fi, the MQTT protocol must be applied to publish all messages and to receive feedback messages among the cloud platforms, RFID controller, and health devices, as shown in Figure 3. Messaging dispatch and retrieval can be performed from and to any place and between any two machines via the Internet. This architecture enhances the scalability and extensibility of the system to other ISs and software components.

### 2.3. Hardware and Software

**Figure 4:**
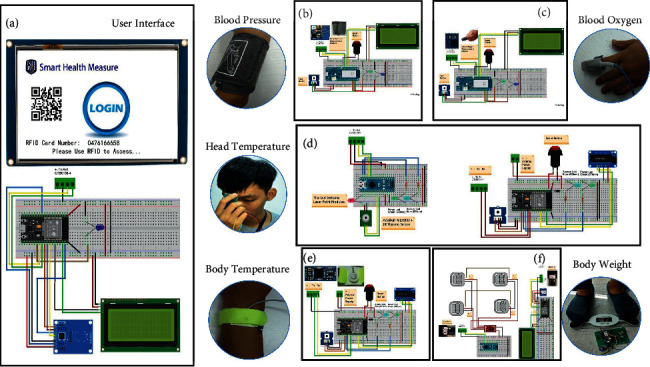
Electronic circuits of all devices discussed in this paper. (a) shows the RFID controller circuits for the MCU (ESP32), a 7″ TFT touch screen (Nextion USART HMI) manufactured by ITEAD STUDIO, and an RFID reader (MFC522) manufactured by NXP Semiconductors. (b) shows the circuits of the blood pressure device for the MCU (Arduino MKR1000), the LCD2004 display, the blood pressure measurement module with an embedded ARM-based MCU modified from a commercial blood pressure product, a user-triggered push button, and a status-indicator light-emitting diode (LED). (c) shows the circuits of the oxygen device for the MCU (Arduino MKR1000), the LCD2004 display, the pulse oximeter with an embedded ARM-based MCU as its controller, a user-triggered push button, and a status-indicator LED. (d) shows the circuits of the head temperature device, which are separated into two parts to include the whole device. The first part is the head temperature probe, which includes the MCU (Arduino Nano), the IR thermo sensor (Nextion, model: LX90614), manufactured by Adafruit Industries, and the status-indicator LED. The second part consists of the controllers, which include an MCU (ESP32), a 0.96″ OLED display manufactured by Seed Technology Co., Ltd., a user-triggered push button, and a status-indicator LED. (e) shows the circuits of the body temperature device, which include the MCU (ESP32), a 0.96″ OLED display manufactured by Seed Technology Co., Ltd., the high-precision thermistor integrated with a 24-bit ADC developed by this research, a user-triggered push button, and a status-indicator LED. (f) shows the circuits of the body-scale device, which are separated into two parts to include the whole device. The first part is the weight detector, which includes the MCU (Arduino Nano); four load cells (maximum of 50 kgW each) combined with a Wheatstone bridge as the weight sensor, which is located under tempered glass to support a human standing on it; and an RF transmitter module (model: MUART0-B) to send data to the weight controller, manufactured by Sunplus Innovation Technology Inc. in Taiwan. The second part is the weight controller, which includes an MCU (ESP32); an LCD2004 display; an RF transmitter module (model: MUART0-B) as the data receiver for the weight detector, manufactured by Sunplus Innovation Technology Inc. in Taiwan; a user-triggered push button; and a status-indicator LED.

Figure 4 shows the hardware circuits of all health devices and the RFID controller, which is a compatible Mifare 13.56 MHz. First, we used an ESP32 (developed and manufactured by Espressif Systems Co., Ltd., Shanghai) and an Xtensa® 32-bit LX6 dual microprocessors with a clock frequency from 80 MHz to 240 MHz, a 2.4 G Wi-Fi 802.11 b/g/n connection capability and a 4 M flash memory (maximum 16 MB flash), serial peripheral interface (SPI) or 8 MB external SPI SRAM as the MCU to control the RFID reader (MFRC522, manufactured by NXP Semiconductors, Eindhoven, Netherlands) for access via user RFID cards. A liquid-crystal display (LCD) (type: LCD 2004 with an integrated circuit (I^2^C)) interface is attached to the MCU as a machine status display to help developers debug the inner variables in the kernel system. Additionally, a Nextion USART HMI TFT-7″ screen (model: TJC8048T070_011) manufactured by ITEAD STUDIO is employed as a touchscreen to display user information and to provide an HMI to enable the implementation of all procedures (e.g., assigning a health device to obtain measurements) and display visualized feedback on a TFT screen, as shown in Figure 4(a).

The RFID controller proposed in this paper uses an MQTT broker to publish information and subscribe to a specific topic with various commands. Additionally, the broker can send requests and receive measurement results via the Internet to resolve the heterogeneous hardware and software gaps based on an IoT architecture. This controller helps different pieces of hardware and software collaborate without the need to upgrade or adapt the system architecture.

Next, we designed a blood pressure monitoring device to detect the diastolic and systolic blood pressure of clients, as shown in Figure 4(b). The blood pressure index is a critical factor for stroke or diabetes. Therefore, we used the Arduino MKR1000 as the MCU to control the blood pressure measurement module, with an embedded ARM-based MCU as the controller (model DLCK365; the DC power is 6 V, the sampling resolution is 0.1 kPa (1 mmHg), and the blood measuring range is 0∼39.9 kPa (299 mmHg), with an error rate under ±0.4 kPa (3 mmHg) and a heart rate measuring range of 40∼180 bpm). All electronic parts were assembled by SMT and designed and developed by Dongguan Richtek Electronics Co., Ltd., located in Guangzhou, China. This device was modified from a commercial blood pressure product to detect each user's blood pressure and to display the sensor results on an I2C LCD2004 display after the user pushes a trigger button.

The blood pressure device also incorporates an MQTT broker that subscribes to a specific topic with certain commands to measure blood pressure and heart rate. Then, it transmits information by publishing the sensor data for any person or machine with the appropriate access credentials.

Next, we designed a blood oxygen device to continuously detect clients' blood oxygen level and heart rate, as shown in Figure 4(c). Specifically, we used the Arduino MKR1000 as the MCU to control a pulse oximeter with an embedded ARM-based MCU as its controller (model DLCK365; the DC power is 3.5∼5 V, the heart rate measuring range is 40∼180 bpm, and all electronic parts were assembled by SMT and designed and developed by Dongguan Richtek Electronics Co., Ltd., the headquarters of which are located in Guangzhou, China). This device is used to measure the blood oxygen level and display the sensor results on an I2C LCD2004 display after the user pushes a trigger button.

The blood oxygen device also uses an MQTT broker that subscribes to a specific topic with certain commands to measure the blood oxygen level and heart rate. The relevant information is transmitted by publishing the sensor data to the MQTT broker for any person or machine with the appropriate access credentials.

Subsequently, we designed a head temperature thermal scanning device to detect clients' core body temperature. In this approach, a long-distance temperature sensor is necessary, as shown in Figure 4(d). Thus, we chose board-mounted temperature sensors (model number MLX90614ESF-DCI, which has an accuracy of ±0.5 C and a working voltage of 2.6∼3.6 V DC; the output data are digital signals with a specific protocol, and the sensors were designed and manufactured by Melexis, Ypres, Belgium), but the ESP32 could not activate them. Hence, we separated the sensor processing and system control parts. First, we connected an Arduino Nano to the MLX90614ESF-DCI device to perform temperature-driven sensing with request commands via a universal asynchronous receiver/transmitter (UART) from the system control. The system control uses an ESP32 as the MCU to take advantage of the 2.4 G Wi-Fi 802.11 b/g/n connection capability and to send sensor data via a DB agent (written with a personal home page/form interpreter (PHP/FI) running on the cloud platform) as DB middleware. The temperature sensor is controlled by sending request commands and receiving results via the UART. We also added a minidisplay (a 0.96″ organic light-emitting diode (OLED) display, SKU: 104030008, manufactured by Seed Technology Co., Ltd., China) to offer users instant visualization.

The head temperature device proposed in this paper also uses an MQTT broker that subscribes to a specific topic with given commands to measure the head temperature. The relevant information is transmitted by the broker by publishing the sensor data for any person or machine with the appropriate access credentials.

Next, we designed a body temperature device for people who require continuous temperature monitoring over a long period, such as children with a high fever or bedridden patients, as shown in Figure 4(e). Compared with the head temperature device, the body temperature device is faster and more accurate, making it more suitable for the abovementioned clients. We also used an ESP32 as the MCU to control the high-precision thermistor, which is a high-accuracy thermal temperature sensor integrated with a 24-bit analog-to-digital converter (ADC) developed for body temperature sensing with a sampling rate of 2 seconds/time. All functions and specifications were followed by GB/T 21416-2008 (GBT 21416-2008); the measuring range is 25∼45°C, the resolution is 0.01°C, the error rate is under 0.1°C, and the working voltage is 2.0∼5.0 V DC. Thus, clients can obtain one measurement or continuous measurements and then receive the results on an OLED display (manufactured by Seed Technology Co., Ltd.).

The body temperature device also uses an MQTT broker that subscribes to a specific topic with given commands to measure the head temperature. The relevant information is transmitted by the broker by publishing the sensor data for any person or machine with the appropriate access credentials.

We designed a body weight measurement device to detect clients' body weight in an attempt to reduce users' caloric intake and balance their diets, as shown in Figure 4(f). In the research stage, we redesigned a commercial body-scale device with a maximum capacity of 150 kg and a resolution of 100 g that was designed and manufactured by SENCOR via reverse engineering technology. We separated the body-scale device into two parts to integrate the whole device within a limited space, and we deployed a printed circuit board (PCB) layout and created a new mechanism and shell. The first part consists of a weight detector, which uses an Arduino Nano connected to four load cells (with a maximum of 50 kgW for each cell) as the MCU combined with a Wheatstone bridge for weight sensing signals. A 24-bit ADC conversion chip (HX711) was connected under tempered glass to support a human standing on it, and an RF transmitter module (model: MUART0-B, manufactured by Sunplus Innovation Technology Inc., Taiwan) was used as a bridge to establish communication among the weight controllers.

The second part of the device is a weight controller that uses an ESP32 as the MCU to operate the RF transmitter module (model MUART0-B; manufactured by Sunplus Innovation Technology Inc., Taiwan) as a data receiver to establish a communication bridge among the weight detectors during weighing. Here, feedback is provided by publishing the sensor data for access by any person or machine that needs the measured data. The body weight device also uses an MQTT broker that subscribes to a specific topic with given commands to measure body weight and heart rate; the relevant information is transmitted by the broker by publishing the sensor data for any person or machine with the appropriate access credentials.

## 3. Results and Discussion

The SHMS can display users' health profiles on the 7″ TFT touch screen of the RFID controller, as shown in Figure 3, when they use their health devices for measurement. Another choice is integrated visualization on the cloud platform, which can synchronously offer detailed visualized results to family members, the attending physician, the medical clinic, etc.

**Figure 5:**
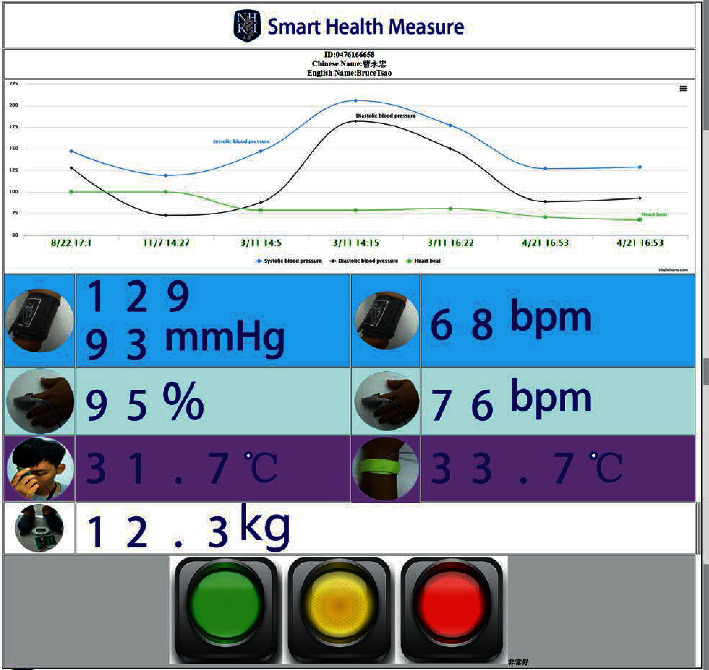
User profiles and physiological health parameters (essential vital signs) kept in the cloud platform developed in this paper. First, users access the website through a browser with their health ID card. The website generates visualized reports based on the measured data and displays a chart of data collected over the previous seven days to visualize the blood pressure trend. Other data can also be displayed side by side. User health is indicated by a red/yellow/green light indicator similar to a traffic signal for analysis and is then summarized from the user profiles and physiological health parameters. Users can also click on icons under the chart in Figure 5 to view the detailed data as a separate chart.

**Figure 6:**
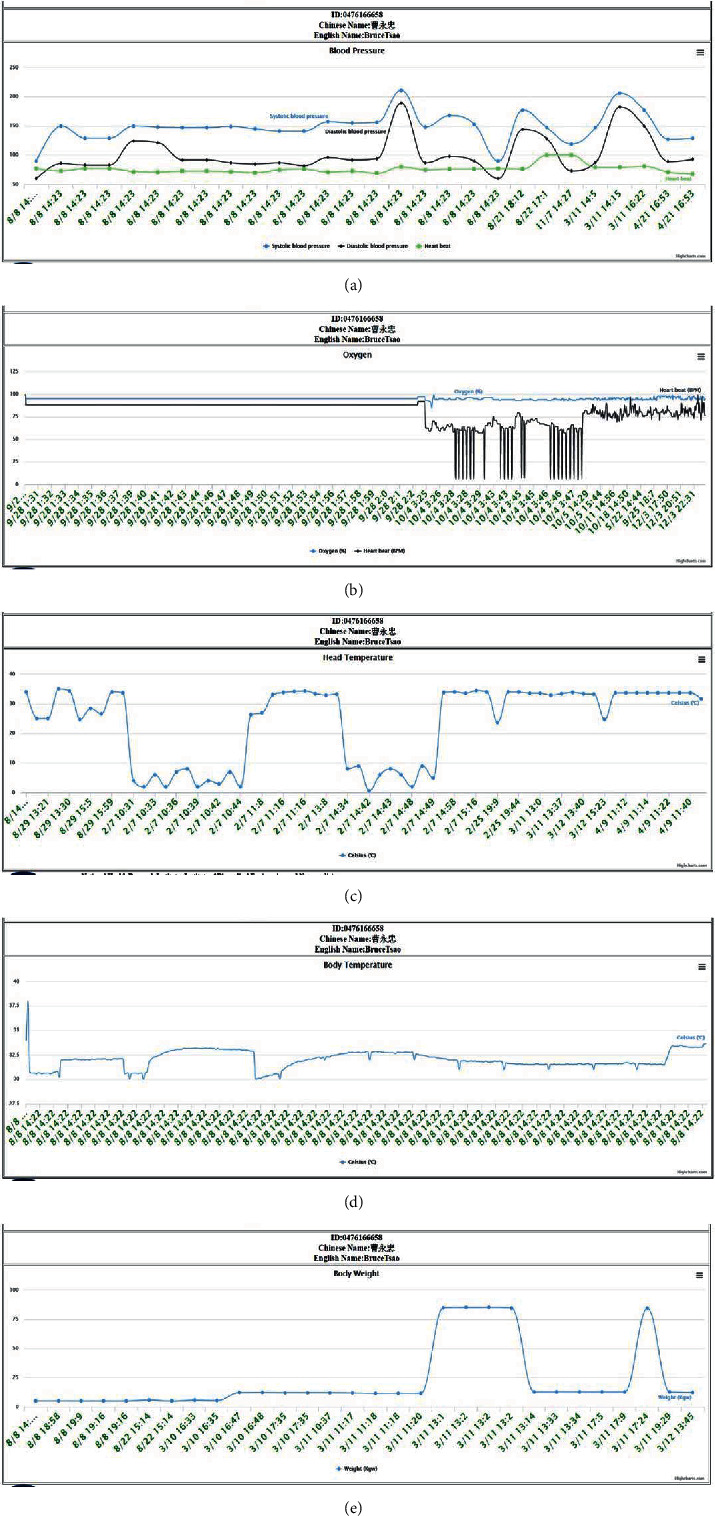
Detailed data displayed after clicking on the icon are shown in Figure 5 and presented as charts. These detailed charts are generated from the cloud platform provided by the SHMS. (a) shows the blood pressure results, including the blood pressure itself (systolic blood pressure and diastolic blood pressure) and the heart rate (maximum of 120 records), produced by our blood pressure device. Clients can learn the circulation status of the whole body, whether their blood pressure status is good or poor, and when hypertension occurs. (b) shows the blood oxygen results, including the blood oxygen level and heart rate (maximum of 120 records), obtained with the blood oxygen device. These results help clients understand the final blood circulation status and the circulation status of the whole body, which indicates whether their blood circulation is good or poor. (c) shows the head temperature results from thermal temperature scans (maximum of 120 records), which can be employed to detect and record body temperature in a contactless way. (d) shows the body temperature results from the elbow (maximum of 120 records) produced by our body temperature device. This device can continue to detect and record body temperature at intervals of a few seconds; thus, it can be applied in unhealthy conditions and can provide data that can be displayed in a long-term continuous temperature chart for the same interval. (e) shows the user weight results (maximum of 120 records) produced by our body weight device, and clients can determine whether they need to improve their health based on, e.g., their BMI. The measured body weight can also indicate blood pressure changes when the body weight changes over a long duration.

Users can use any computer-based device with any browser to access the website, on which they can choose their ID/name or access the RFID card with an RFID reader connected to their device to query their most recent health status, as shown in Figure 5. In addition, if users wish to view detailed information, they can click on the circular icon in front of each health value, and the website will display the corresponding chart, as shown in Figure 6.

This mechanism can be used to notify clients' families or medical personnel when clients who commonly forget daily tasks (e.g., keeping health records) fail to take measurements on time [34]. Of course, clients' family members or medical personnel can also obtain detailed information with this mechanism to understand relevant trends and relationships, as shown in Figure 6, and to establish helpful diagnoses to prevent illness. A novel feature of the SHMS is that each detailed chart, as shown in Figure 6, can be downloaded as raw data in comma-separated value (CSV)/XLS format or saved as an image in JPG format/PNB format/portable document format (PDF) by clicking the upper-right icon in the chart with no extra effort from developers due to the graphical user interface (GUI) technology used (supported by Highsoft, Norway).

Each subfigure describes different health indexes.  Figure 6(a) shows the chart of a client's blood pressure (systolic blood pressure and diastolic blood pressure) and heart rate (maximum of 120 records) to reveal his or her circulation status. The long-term chart indicates whether a user's blood pressure status is good or poor and whether hypertension is present.

Figure 6(b) shows the chart of the blood oxygen results (maximum of 120 records) to reveal the current blood circulation condition. The long-term chart can indicate whether a user's blood circulation is good or poor.  Figure 6(c) shows the chart of the head temperature results (maximum of 120 records) to reveal potential variations in body temperature. This chart can help clients' family members understand moderate-term changes in client temperatures. With the chart, some symptoms can be detected quickly.  Figure 6(d) shows the chart of continuously collected body temperature data for clients who were sick in an observation room (maximum of 120 records). The corresponding health device, which is similar to a belt, can automatically detect a client's temperature. Thus, long-term body temperature data can easily be collected and displayed to reveal a client's stable body temperature or unpredictable changes with exact date-time tags.  Figure 6(e) shows a chart of user weight results (maximum of 120 records) that can be utilized by clients to determine how to improve their health based on, e.g., body mass index (BMI) or by referring to their blood pressure changes, as shown in Figure 6(a), when their body weight changes over a long period. Therefore, the long-term charts based on data from different health devices can be combined for viewing based on specific goals, as shown in Figures 6(a)–6(e).

The external middleware used for data exchange in the SHMS can reduce handwriting and error problems encountered during diagnosis and medical treatment. Translating handwritten records to digital ones is difficult and cumbersome for patients and their attending physicians, and handwritten records often require much effort to read. Therefore, digital health devices and synchronized data sharing among patients, family members, and doctors can be simplified by implementing the abovementioned processes, and quicker responses to patient medical records can be obtained than ever before. To address these critical issues, the newly proposed IoT-based technology with an MQTT broker for both publishing and subscribing to topics can be applied to the SHMS. However, data exchange from legacy ISs will incur massive costs and effort in terms of redesign or migration. Thus, IS updating may be incomplete, and certain ISs may not be suitable for the modern age. For a better solution, in this paper, we propose a new IoT-based architecture to enable SHMS data control, transmission, and retrieval from other devices that are not designed or developed here.

The SHMS architecture can enhance FDA-approved health devices located in a hospital or in a home. With support from the MQTT broker, health device usage and omnipresent monitoring can be made accessible to family members via easy-access middleware based on the autonomous communication of the SHMS. Another excellent benefit is that clients can help create long-term digital records, which can be retrieved from the MIS with an MQTT broker by easily subscribing to a specific topic without being deeply involved in the SHMS. Moreover, long-term digital records can be easily read and analyzed [35].

In addition to monitoring patients' health, there are numerous different regions in which IoT gadgets can be helpful in emergency clinics. IoT gadgets labeled with sensors are utilized to follow clinical hardware, such as wheelchairs, defibrillators, nebulizers, oxygen siphons, and other monitoring equipment. The organization of clinical staff in various areas can likewise be continuously investigated. The spread of contamination is a major concern for patients in medical clinics. IoT-empowered cleanliness-checking gadgets help patients avoid infection. IoT gadgets likewise help manage resources, such as drug stock control, and environmental observations, for example, actually viewing refrigerator temperature and stickiness and room temperature control.

These new technologies can improve medical management. If patient history information can be transmitted and shared in a timely manner, it will promote medical efficiency and effectively improve the quality of medical monitoring. Therefore, information transmission and transparency are very important parts of medical management. Through the IoT, the inefficiency of procedures for obtaining information in medical institutions can be reduced, and medical staff can provide patients with suitable treatment strategies in a timely manner and reduce the sequelae caused by delayed treatment. For patients, the waiting time can be reduced, thus preventing uncertainty about their future medical care. In addition, through the mechanism shown in Figure 6, it is possible to monitor and remind patients to measure related indicators to improve the completeness of information aggregation. Overall, if patient information can be effectively transmitted, the quality of medical care will be improved. Accurate and efficient medical treatment will create positive perceptions in patients and provide affirmation of the quality of medical institutions, thus increasing patients' trust. Shared medical records technology provides a timely, flexible, and information integration mechanism for medical management. It can provide managers with analyses that support decision-making and medical quality monitoring indicators to improve management quality and timeliness.

In summary, the developed SHMS applies many IoT-related technologies, including an embedded system, an MQTT broker, bioinformation technology, sensor technology, distributed system technology, web technology, cloud computing, and visualization techniques, to collaboratively build middleware and transmit bidirectional information among devices. This approach can solve the closeness issues of MISs and reduce the heavy burden on clients, attending physicians, and family members associated with sharing health data in an MIS. Remote monitoring can enable the transmission of uninterrupted notifications regarding patients' daily health parameters, such as temperature, body weight, blood pressure, and blood glucose level. In addition, with the SHMS, the costs of these health devices will be reduced, regardless of whether they are home-use sensors, commercial sensors, or FDA-approved sensors.

## 4. Conclusions

In this study, we discuss an SHMS that uses an RFID controller as the central MCU via an MQTT broker to transmit control commands and provide feedback results. This approach simplifies and accelerates the communication of information via a global standard MQTT protocol, sending data in less than 1 second in an Internet environment, as demonstrated over more than six months of testing. With the QoS of the MQTT broker, the error ratios are less than 1% on average over one thousand tests, and the SHMS is highly stable in a Wi-Fi environment despite basic wireless networking problems. The developed blood pressure device, which was tested over six months with random sampling, has an error ratio of less than ±2% on average, based on the results of a commercial automatic blood pressure monitoring device. The developed blood oxygen device, which was tested over six months with random sampling, has an error ratio of less than ±1.5% on average, based on the results of a commercial oximeter. The proposed head temperature device has an error ratio of less than ±0.8°C on average, based on the results of a commercial thermal scanner, and the proposed body temperature device has an error ratio of less than ±1.2°C on average, based on the results of a commercial thermal scanner; both proposed devices were tested over six months with random sampling. The body-scale device, which was tested over six months with random sampling, has an error ratio of less than ±0.8 kgW on average, based on the results of a commercial body scale. The abovementioned experimental records indicate that all health devices used in the SHMS are reliable and accurate.

The web page of the cloud platform, as shown in Figure 5, presents trends over seven days or sessions based on client blood pressure and heart rate data, allowing clients' doctors and family members to remotely view the clients' vital parameters with a browser via a desktop personal computer (PC) or mobile device (smartphone). In addition, because these blood pressure and heart rate trends may reveal initial symptoms that may indicate chronic deterioration, clients' doctors can click on the circular icon in front of each health value to explore the health data in detail and make advanced diagnoses. The SHMS also asynchronously transmits all health sensor data to the MQTT server, which allows the MIS to subscribe to or be added to DB systems to merge patient medical records. This mechanism solves the traditional data porting problem, reduces the effort required to interpret records written by hand, and prevents misjudgment issues as a result of illegible writing.

The presentation of diagnoses as traffic signals at the bottom of clients' health web pages, as shown in Figure 5, indicates each client's level of exercise or standard of living, including food intake, medicine consumption, consumption of food or drink with high fat or sugar content, and sleep [36]. In the future, the SHMS will apply artificial intelligence (AI) technology to enhance these functions [36]. These functions also provide instantaneous downloads of raw data, including heart rate data, blood oxygen levels, blood pressure data, body weight data, and continuously monitored body temperature data, in the popular CVS/XLS format when the user clicks on the upper-right icon in any chart. This information can then be sent to other applications, such as Excel. In the future, the SHMS will be extended for multiple patients and new plug-in health devices following the standard MQTT protocol with no major changes, and home-use health devices and FDA-approved medical equipment can be used in any related MIS.

## Data Availability

Data will be provided on request through the corresponding author of this article.
